# Comparative Evaluation of Oral Brush Cytology and Toluidine Blue Staining in the Detection of Oral Epithelial Dysplasia: A Prospective Clinical Study

**DOI:** 10.7759/cureus.110427

**Published:** 2026-06-07

**Authors:** Ivanpreet Kaur, Angshuman Saha, Aditi Das, Suraiya Khan, Anju Gopinathan T, Sneha S Vijayashekar

**Affiliations:** 1 Department of Pathology, Government Medical College and Rajindra Hospital, Patiala, IND; 2 Department of Pathology, KPC Medical College and Hospital, Kolkata, IND; 3 Department of Pathology, Gouri Devi Institute of Medical Sciences and Hospital, Durgapur, IND; 4 Department of Public Health, Johns Hopkins Bloomberg School of Public Health, Baltimore, USA; 5 Department of Oral and Maxillofacial Surgery, Government Dental College and Hospital, Ahmedabad, IND; 6 Department of Oral Pathology and Microbiology, Farooqia Dental College and Hospital, Mysuru, IND

**Keywords:** oral biopsy, oral brush cytology, oral squamous cell carcinoma, potentially malignant oral disorders, toluidine blue

## Abstract

Introduction: Early detection of potentially malignant oral disorders and oral squamous cell carcinoma plays a critical role in reducing disease progression and improving patient survival. The present study aimed to comparatively evaluate the diagnostic efficacy of oral brush cytology and toluidine blue staining in detecting dysplastic and malignant oral lesions using histopathological examination as the gold standard.

Materials and Methods: This prospective clinical study included 150 participants with clinically suspicious oral mucosal lesions. Oral brush cytology and toluidine blue staining were performed prior to incisional biopsy in all participants. Histopathological examination served as the reference standard for confirmation of dysplasia and malignancy. Diagnostic performance parameters, including sensitivity, specificity, positive predictive value (PPV), negative predictive value (NPV), diagnostic accuracy, area under the receiver operating characteristic curve (AUC), and Cohen’s kappa coefficient, were calculated and compared. Statistical analysis was performed, and p < 0.05 was considered statistically significant.

Results: Histopathological examination revealed dysplastic or malignant changes in 102 lesions (68.0%). Oral brush cytology demonstrated sensitivity of 87.3%, specificity of 81.3%, PPV of 90.8%, NPV of 75.0%, and diagnostic accuracy of 85.3%. Toluidine blue staining showed sensitivity of 82.4%, specificity of 70.8%, PPV of 85.7%, NPV of 65.4%, and diagnostic accuracy of 78.7%. Oral brush cytology demonstrated significantly higher AUC (0.843 vs. 0.766; p = 0.038) and greater agreement with histopathology (κ = 0.672 vs. 0.527; p = 0.041) compared with toluidine blue staining. Combined positivity of both tests further improved agreement with histopathology (κ = 0.718).

Conclusion: Within the limitations of the study, both oral brush cytology and toluidine blue staining demonstrated satisfactory diagnostic utility for identifying dysplastic and malignant oral lesions. However, oral brush cytology exhibited superior overall diagnostic performance and agreement with histopathology, suggesting its greater reliability as a non-invasive adjunctive screening tool for early detection of oral epithelial dysplasia and malignancy.

## Introduction

Oral cancer represents a significant global health burden, with GLOBOCAN 2020 reporting approximately 377,000 new cases and 177,000 deaths annually, with the highest incidence concentrated in South and Southeast Asian countries [1]. India alone accounts for nearly one-third of the total global burden of oral cancer. The majority of oral cancers arise from oral potentially malignant disorders (OPMDs), a heterogeneous group of mucosal conditions including leukoplakia, erythroplakia, oral submucous fibrosis, and oral lichen planus that carry an elevated risk of malignant transformation [2]. Lesions with dysplastic features have been categorized under OPMDs, which are assumed to have a high risk of malignancy, and early detection, prevention, and treatment of OPMDs are needed to prevent their malignant transformation into oral cancer [3]. Oral epithelial dysplasia (OED) serves as the primary histopathological indicator of this malignant potential, and its timely and accurate identification is therefore central to improving patient survival and treatment outcomes [4].

The conventional gold standard for diagnosing OED remains incisional biopsy followed by histopathological examination; however, its invasive nature, patient anxiety, and requirement for trained personnel limit its suitability as a routine or mass-screening tool. Detection of premalignant lesions of the oral mucosa allows for treatment that may be sufficiently early to prevent their progression to invasive carcinoma, although techniques for supplementing clinical examination have variable false-negative rates [5]. This clinical reality has driven considerable research interest in adjunctive, non-invasive, or minimally invasive chairside methods capable of reliably triaging suspicious lesions before committing to formal biopsy [6].

Oral brush cytology has emerged as one such adjunct, offering a minimally invasive means of sampling the full transepithelial cell population of a lesion. Brush biopsy is a simple, relatively inexpensive, high-sensitivity, risk-free method of screening for cancer and serves as an aid to clinical examination [7]. Studies employing liquid-based oral brush cytology have reported accuracy values exceeding 91%, with sensitivity and specificity of 79.23% and 94.81%, respectively, supporting its role as a reliable adjunct to surgical biopsy in the diagnosis of OPMDs [7,8]. Toluidine blue staining represents a complementary approach, exploiting the increased nucleic acid content of dysplastic cells. Since dysplasia and carcinoma in situ contain considerably more DNA and RNA than the surrounding normal epithelium, toluidine blue selectively stains these acidic tissue components, and studies assessing its performance have reported sensitivity and specificity ranging from 93.5% to 97.8% and 73.3% to 92.9%, respectively [9,10]. Despite these promising individual performances, published data on the direct comparative diagnostic accuracy of brush cytology versus toluidine blue staining for OED detection remain limited and inconsistent.

While both adjunct methods identified 92% of carcinoma in situ and squamous cell carcinoma as confirmed by histopathology, compared with only 62% identified by clinical findings alone, the sensitivity and specificity profiles of the two techniques differ, and their relative merits in detecting varying grades of epithelial dysplasia have not been conclusively established [11]. Furthermore, existing studies vary considerably in lesion selection criteria, staining protocols, and cytological interpretation criteria, making inter-study comparisons unreliable. The primary objective of the present study was to compare the diagnostic efficacy of oral brush cytology and toluidine blue staining in detecting OED and malignancy using histopathological examination as the reference standard. Secondary objectives included determining and comparing the sensitivity, specificity, positive predictive value (PPV), negative predictive value (NPV), and diagnostic accuracy of both diagnostic modalities; evaluating their overall diagnostic performance using receiver operating characteristic (ROC) curve analysis and area under the curve (AUC) values; assessing their agreement with histopathological findings using Cohen's kappa coefficient; and exploring the potential complementary value of combining both non-invasive techniques.

## Materials and methods

Study design and setting

The present study was designed as a prospective clinical study conducted in the Department of Pathology at Gouri Devi Institute of Medical Sciences and Hospital, Durgapur, India, between October 2023 and March 2024. The study protocol was conducted in accordance with the principles of the Declaration of Helsinki for biomedical research involving human participants. Ethical clearance was obtained from the Institutional Ethics Committee prior to the commencement of the study (Approval No.: GIMSH/R.O/23/18). Written informed consent was obtained from all participants prior to their enrollment in the study.

Study population and sampling

A total of 150 participants presenting with clinically suspicious oral mucosal lesions were included in the study using a consecutive sampling technique. Adult patients aged 18 years and above with lesions clinically suggestive of OPMDs or early malignant transformation, including leukoplakia, erythroplakia, verrucous lesions, ulcerative lesions, and oral submucous fibrosis, were considered eligible for inclusion. Patients willing to undergo both non-invasive diagnostic procedures and confirmatory incisional biopsy were recruited after obtaining informed consent. Patients with a previous history of radiotherapy or chemotherapy for head and neck malignancies, known bleeding disorders, anticoagulant therapy, advanced necrotic lesions unsuitable for brush sampling, and pregnant or lactating women were excluded from the study. Detailed demographic history, tobacco habits, alcohol consumption, lesion characteristics, site, size, and clinical appearance were recorded using a standardized case history proforma.

Sample size estimation

Sample size estimation was performed using the G*Power software (version 3.1.9.7, Heinrich Heine University, Düsseldorf, Germany). Based on previous studies demonstrating an approximate sensitivity of 85% for non-invasive diagnostic tests in detecting oral dysplastic lesions, the minimum required sample size was calculated using a one-sample proportion test with a 95% confidence interval, 80% statistical power, and an alpha error of 5% [12]. The minimum sample size was estimated as 120. To compensate for inadequate or inconclusive samples, an additional 20% were added, resulting in a final sample size of 150 participants.

Clinical examination procedure

All participants underwent comprehensive oral examination under adequate illumination using sterile mouth mirrors and gauze. The clinical characteristics of lesions, including site, size, color, surface texture, and margins, were recorded. Clinical photographs were obtained before performing the non-invasive diagnostic procedures. All index tests were carried out prior to incisional biopsy to avoid tissue contamination or procedural bias. The examiner performing the non-invasive procedures was blinded to the histopathological findings, while the histopathologist and cytopathologist were blinded to the clinical and adjunctive test results.

Oral brush cytology procedure

**Figure 1:**
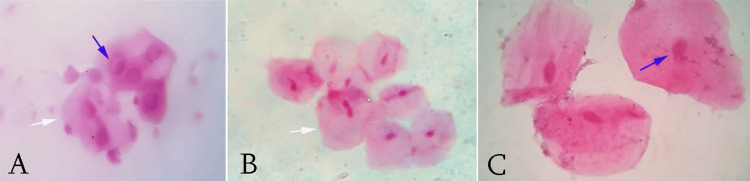
Papanicolaou stained cytological smears showing (A) epithelial cells with marked nuclear pleomorphism (blue arrow) and dysplastic features (white arrow) at 40× magnification; (B) epithelial cells exhibiting mild pleomorphism (white arrow) at 40× magnification; and (C) normal epithelial cells without dysplastic changes and with normal nuclear morphology (blue arrow) at 100× magnification.

Oral brush cytology sampling was performed using a sterile cytobrush (Orcellex Brush; Rovers Medical Devices B.V., Oss, the Netherlands). The brush was rotated firmly over the most suspicious area of the lesion approximately 5-10 times until pinpoint bleeding was observed, thereby ensuring transepithelial cell collection. The harvested cellular material was immediately smeared evenly onto clean glass slides and fixed in 95% ethanol. The slides were subsequently stained with Papanicolaou stain and evaluated under light microscopy by an experienced oral cytopathologist blinded to the clinical diagnosis and histopathological outcome [13]. Cytological findings were categorized as inadequate, negative for dysplasia, atypical, suspicious for malignancy, or positive for malignancy (Figure 1). For statistical analysis, the atypical, suspicious, and malignant categories were considered positive for dysplastic or malignant changes.

Toluidine blue staining procedure

Toluidine blue staining was performed using a 2% toluidine blue vital staining solution (Sisco Research Laboratories Pvt. Ltd., Mumbai, India). Initially, the lesion area was rinsed with 1% acetic acid solution for 20 s to remove surface debris and salivary contamination. Toluidine blue solution was then applied over the lesion using a sterile cotton applicator and allowed to remain in contact with the mucosa for approximately 20-30 s. The excess dye was subsequently removed by rinsing with 1% acetic acid, followed by distilled water [9]. Lesions demonstrating dark royal blue retention were considered positive or suspicious, whereas those showing faint or no dye retention were considered negative. Staining was interpreted by the principal investigator before the biopsy.

Histopathological examination

Following the completion of both non-invasive procedures, an incisional biopsy was performed from the most clinically suspicious region of the lesion using a sterile scalpel blade or a punch biopsy instrument. Tissue specimens were immediately fixed in 10% neutral-buffered formalin and processed using standard paraffin embedding. Sections of 4-5 µm thickness were prepared and stained with hematoxylin and eosin for microscopic evaluation [14].

The lesions were classified as normal epithelium, hyperkeratosis or epithelial hyperplasia, mild dysplasia, moderate dysplasia, severe dysplasia or carcinoma in situ, or invasive squamous cell carcinoma. For the diagnostic performance analysis, lesions showing mild dysplasia or higher grades were considered histopathologically positive (dysplastic group), whereas normal and hyperkeratotic lesions were considered negative (non-dysplastic group).

Outcome measures

The primary outcome measures of the study included sensitivity, specificity, PPV, NPV, and diagnostic accuracy of both the groups in detecting dysplastic and malignant oral lesions. Secondary outcome measures included assessment of agreement between non-invasive tests and histopathology using Cohen’s kappa coefficient (κ), as well as evaluation of the AUC for the ROC of overall diagnostic performance.

To maintain methodological blinding and minimize observer bias, oral brush cytology evaluation was independently performed by the first author (IK), toluidine blue staining assessment by the second author (AS), and histopathological examination by the sixth author (SSV), who remained blinded to the adjunctive test findings. All the authors belonged to different institutions for independent assessment and to reduce observer bias. Patient recruitment, clinical examination, and procedural coordination were conducted by the third author (AD), while the fourth and fifth authors (SK and AGT, respectively) independently performed statistical analysis and data verification and were provided with coded data. This division of responsibilities across participating institutions helped ensure objectivity, blinded assessment, and methodological rigor throughout the study.

Statistical analysis

All collected data were analyzed using IBM SPSS Statistics for Windows, Version 25.0 (Released 2017, IBM Corp., Armonk, New York). Descriptive statistics were expressed as frequencies and percentages. Diagnostic performance parameters, including sensitivity, specificity, PPV, NPV, and overall diagnostic accuracy, were calculated with 95% confidence intervals using the Wilson score method. The agreement between non-invasive diagnostic methods and histopathological findings was assessed using Cohen’s kappa coefficient (κ). Paired comparisons between oral brush cytology and toluidine blue staining were performed using the McNemar’s test. ROC curves were generated and the AUC values were compared using DeLong’s method. Statistical significance was set at p < 0.05.

## Results

In total, 150 participants with clinically suspected oral mucosal lesions were included in this study. Histopathological examination is considered the gold standard for confirming the presence of dysplastic and malignant lesions. The diagnostic performance of oral brush cytology and toluidine blue staining was evaluated and compared using various statistical parameters.

Sociodemographic and clinical characteristics

The sociodemographic and clinical profiles of the participants are presented in Table 1. The majority of the participants belonged to the 46-60 years age group, followed by the 31-45 years age group. The study sample consisted of 91 men (60.7%) and 59 women (39.3%). Smoking was the most prevalent habit, followed by smokeless tobacco use and alcohol consumption. Leukoplakia was the most common lesion type, and the buccal mucosa was the most frequently affected site.

**Table 1:** Sociodemographic and clinical characteristics of the study participants (n = 150). Data are presented as frequency and percentage; descriptive statistics are used for analysis, and n = number of participants.

Characteristic	Category	n (%)
Age (years)	18-30	22 (14.7%)
	31-45	48 (32.0%)
	46-60	54 (36.0%)
	≥61	26 (17.3%)
Sex	Male	91 (60.7%)
	Female	59 (39.3%)
Habit	Smoker	67 (44.7%)
	Smokeless tobacco	48 (32.0%)
	Alcohol use	35 (23.3%)
	No habit	18 (12.0%)
Lesion type	Leukoplakia	72 (48.0%)
	Erythroplakia	24 (16.0%)
	Oral submucous fibrosis	18 (12.0%)
	Verrucous lesion	21 (14.0%)
	Ulceration	15 (10.0%)
Lesion site	Buccal mucosa	58 (38.7%)
	Tongue	34 (22.7%)
	Floor of mouth	27 (18.0%)
	Lip/commissure	19 (12.7%)
	Other sites	12 (8.0%)

Histopathological diagnosis distribution

The histopathological diagnoses of all the lesions are summarized in Table 2. Among the 150 lesions examined, 18 (12.0%) showed normal epithelium and 30 (20.0%) demonstrated hyperkeratosis or epithelial hyperplasia. Mild dysplasia was identified in 37 lesions (24.7%), moderate dysplasia in 32 lesions (21.3%), and severe dysplasia/carcinoma in situ in 22 lesions (14.7%). Invasive squamous cell carcinoma was observed in 11 (7.3%) patients. Overall, 102 lesions (68.0%) were categorized as dysplastic or malignant, whereas 48 (32.0%) were considered non-dysplastic.

**Table 2:** Histopathological diagnosis distribution of oral lesions (n = 150). Data are presented as frequency and percentage; histopathological examination is used as the gold standard reference test. CIS: carcinoma in situ.

Histopathological diagnosis	n (%)
Normal epithelium	18 (12.0%)
Hyperkeratosis/Epithelial hyperplasia	30 (20.0%)
Mild dysplasia	37 (24.7%)
Moderate dysplasia	32 (21.3%)
Severe dysplasia/CIS	22 (14.7%)
Invasive squamous cell carcinoma	11 (7.3%)
Dichotomised: Non-dysplastic (Normal + Hyperkeratosis)	48 (32.0%)
Dichotomised: Dysplastic (Mild-Severe + Invasive squamous cell carcinoma)	102 (68.0%)

Diagnostic comparison of oral brush cytology and toluidine blue staining

A comparison of non-invasive diagnostic procedures and histopathology is presented in Table 3. Oral brush cytology correctly identified 89 out of 102 dysplastic lesions, yielding a sensitivity of 87.3%, whereas toluidine blue staining detected 84 dysplastic lesions with a sensitivity of 82.4%. Oral brush cytology demonstrated higher specificity (81.3%) than toluidine blue staining (70.8%). False-positive and false-negative results were lower with oral brush cytology than with toluidine blue staining.

**Table 3:** Comparison of oral brush cytology and toluidine blue staining with histopathology. Data were presented as frequency and percentage; histopathological diagnosis was used as the reference standard; and sensitivity and specificity were calculated for each non-invasive test.

Index test	Reference test	Dysplastic (Histopathology positive)	Non-dysplastic (Histopathology negative)	Row total
Oral brush cytology result	Positive	89 (87.3%)	9 (18.8%)	98 (65.3%)
	Negative	13 (12.7%)	39 (81.2%)	52 (34.7%)
	Column total	102 (100%)	48 (100%)	150 (100%)
Toluidine blue staining result	Positive	84 (82.4%)	14 (29.2%)	98 (65.3%)
	Negative	18 (17.6%)	34 (70.8%)	52 (34.7%)
	Column total	102 (100%)	48 (100%)	150 (100%)

Diagnostic performance analysis

**Figure 2:**
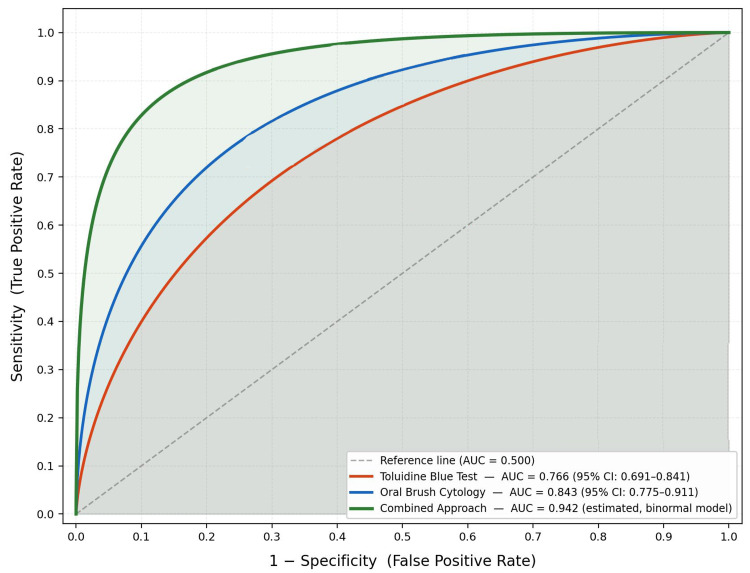
Receiver operating characteristics curve for oral brush biopsy, toluidine blue staining, and combined approach. AUC: area under the curve; CI: confidence interval.

The diagnostic performance parameters for both tests are listed in Table 4. Oral brush cytology demonstrated a higher sensitivity (87.3%), specificity (81.3%), PPV (90.8%), NPV (75.0%), and diagnostic accuracy (85.3%) than toluidine blue staining, which showed a sensitivity of 82.4%, specificity of 70.8%, PPV of 85.7%, NPV of 65.4%, and diagnostic accuracy of 78.7%. The AUC curve (Figure 2) was significantly greater for oral brush cytology (0.843) than for toluidine blue staining (0.766) (p = 0.038). Similarly, Cohen’s kappa coefficient demonstrated significantly better agreement between oral brush cytology and histopathology (κ = 0.672) than toluidine blue staining (κ = 0.527) (p = 0.041).

**Table 4:** Diagnostic performance of oral brush cytology and toluidine blue staining. Data are presented as percentages and coefficients. McNemar’s test is used for paired comparison of proportions, DeLong’s method is used for comparison of ROC curves, and Cohen’s kappa is used for agreement analysis. *p < 0.05 is considered statistically significant. ROC: receiver operating characteristic; κ: kappa coefficient; AUC: area under the curve.

Parameter	Oral brush cytology		Toluidine blue staining		Test value	p-value
	Estimate (%)	95% CI	Estimate (%)	95% CI		
Sensitivity	87.3%	79.2-93.0%	82.4%	73.4–89.3%	χ² = 1.02	0.312
Specificity	81.2%	67.4-91.1%	70.8%	55.9-83.1%	χ² = 1.77	0.184
Positive predictive value (PPV)	90.8%	83.3-95.7%	85.7%	77.0-92.0%	χ² = 1.46	0.226
Negative predictive value (NPV)	75.0%	60.4-86.4%	65.4%	51.0-77.8%	χ² = 1.39	0.237
Diagnostic accuracy	85.3%	78.6-90.6%	78.7%	71.3-85.0%	χ² = 2.10	0.146
AUC	0.843	0.775-0.911	0.766	0.691-0.841	Z = 2.07	0.038*
Cohen's Kappa (κ)	0.672	0.553-0.791	0.527	0.391-0.663	Z = 2.04	0.041*

Subgroup analysis according to lesion type

The subgroup sensitivity analysis according to the lesion type is presented in Table 5. Oral brush cytology consistently demonstrated higher sensitivity than toluidine blue staining across most lesion categories. For leukoplakia lesions, the sensitivities were 86.3% for oral brush cytology and 80.4% for toluidine blue staining. For erythroplakia lesions, the sensitivity was 95.5% and 90.9%, respectively. Lower sensitivities were observed for verrucous lesions in both tests. However, the differences between the two diagnostic methods within individual lesion categories were not statistically significant (p > 0.05).

**Table 5:** Subgroup analysis of sensitivity according to lesion type. Data are presented as percentages; the chi-square test or Fisher’s exact test is used for subgroup comparison, and n = number of dysplastic lesions confirmed by histopathology.

Lesion type	Dysplastic lesions (n%)	Oral brush cytology sensitivity (%)	Toluidine blue staining sensitivity (%)	Test value (χ²)	p-value
Leukoplakia	51 (70.83%)	86.3%	80.4%	0.64	0.391
Erythroplakia	22 (91.67%)	95.5%	90.9%	0.37	0.542
Oral submucous fibrosis	15 (83.33%)	86.7%	80.0%	0.24	0.617
Verrucous lesion	9 (42.85%)	66.7%	55.6%	0.23	0.587
Ulceration	5 (33.33%)	80.0%	80.0%	0.01	1.000
Overall	102 (68%)	87.3%	82.4%	0.95	0.312

Agreement analysis with histopathology

The inter-test agreement with histopathology using Cohen’s kappa coefficient is summarized in Table 6. Oral brush cytology showed substantial agreement with histopathology (κ = 0.672), whereas toluidine blue staining showed a moderate agreement (κ = 0.527). When both non-invasive tests were considered together, agreement improved further (κ = 0.718), indicating substantial concordance with the histopathological diagnosis. All kappa values were statistically significant (p < 0.001).

**Table 6:** Agreement analysis of non-invasive tests with histopathology using Cohen’s kappa. Cohen’s kappa coefficient is used for agreement analysis. Interpretation of kappa values: κ < 0.20 = poor agreement, 0.21-0.40 = fair agreement, 0.41-0.60 = moderate agreement, 0.61-0.80 = substantial agreement, 0.81-1.00 = almost perfect agreement; *p < 0.05 is considered statistically significant.

Test	Cohen’s Kappa (κ)	95% CI	Interpretation	p-value
Oral brush cytology	0.672	0.553-0.791	Substantial	< 0.001*
Toluidine blue staining	0.527	0.391-0.663	Moderate	< 0.001*
Combined (Both positive)	0.718	0.608-0.828	Substantial	< 0.001*

Combined diagnostic approach

Among the 102 histopathologically confirmed dysplastic cases (Table 7), oral brush cytology and toluidine blue showed concordant positive results in 75 (73.5%) cases, while both methods failed to detect dysplasia in four (3.9%) cases. Oral brush cytology alone correctly identified 14 (13.7%) dysplastic cases that were missed by toluidine blue, whereas toluidine blue alone correctly detected nine (8.8%) cases missed by oral brush cytology.

**Table 7:** Contingency table for paired comparison between OBC and TB (McNemar's test) for dysplastic lesions. OBC: oral brush cytology; TB: toluidine blue staining.

Dysplastic (Histopathology positive)	TB Positive (TP)	TB Negative (FN)	Row total
OBC positive (TP)	75 (concordant +)	14 (OBC only correct)	89
OBC negative (FN)	9 (TB only correct)	4 (both wrong)	13
Column total	84	18	102

Among the 48 non-dysplastic cases (Table 8), both methods correctly classified 27 (56.3%) cases and incorrectly classified two (4.2%) cases. Oral brush cytology alone correctly identified 12 (25.0%) non-dysplastic cases compared to seven (14.6%) cases correctly identified only by toluidine blue. Overall, oral brush cytology demonstrated a slightly higher diagnostic yield than toluidine blue in both dysplastic and non-dysplastic lesions.

**Table 8:** Contingency table for paired comparison between OBC and TB (McNemar's test) for non-dysplastic lesions. OBC: oral brush cytology; TB: toluidine blue staining.

Non-dysplastic (Histopathology negative)	TB Negative (TN)	TB Positive (FP)	Row Total
OBC Negative (TN)	27 (concordant -)	12 (OBC only correct)	39
OBC Positive (FP)	7 (TB only correct)	2 (both wrong)	9
Column total	34	14	48

Comparison of the combined diagnostic approach with histopathological findings revealed that 95 (93.1%) of 102 histopathologically confirmed dysplastic cases were correctly identified, whereas 37 (77.1%) of 48 non-dysplastic cases were accurately classified (Table 9). The combined approach produced 11 (22.9%) false-positive and seven (6.9%) false-negative results. Overall, 132 (88.0%) of 150 cases were correctly diagnosed, demonstrating good diagnostic performance and substantial agreement with histopathological assessment for the detection of epithelial dysplasia.

**Table 9:** Contingency table of the combined approach.

Combined approach versus histopathology	Dysplastic (Histopathology positive)	Non-dysplastic (Histopathology negative)	Total
Combined positive	95 (93.1%)	11 (22.9%)	106 (70.7%)
Combined negative	7 (6.9%)	37 (77.1%)	44 (29.3%)
Combined total	102 (100.0%)	48 (100.0%)	150 (100.0%)

## Discussion

The present study comparatively evaluated the diagnostic efficacy of oral brush cytology and toluidine blue staining for detecting dysplastic and malignant oral lesions using histopathology as the reference standard. The findings demonstrated that both techniques showed satisfactory diagnostic performance, although oral brush cytology consistently exhibited superior sensitivity, specificity, diagnostic accuracy, and agreement with histopathology when compared with toluidine blue staining.

In the present study, the majority of participants were men in the age group of 46-60 years, with tobacco-related habits being highly prevalent. These findings are consistent with previously published epidemiological studies reporting a higher incidence of OPMDs and OSCC among middle-aged and elderly men with tobacco exposure [15]. Leukoplakia was the most commonly observed lesion, followed by erythroplakia and verrucous lesions, which is comparable to the distribution reported in studies by Masthan et al. [16] and van der Waal et al. [17], where leukoplakia represented the predominant OPMD evaluated using adjunctive diagnostic methods.

Histopathological examination revealed that 68% of lesions demonstrated dysplastic or malignant changes, indicating a relatively high-risk study population. Mild dysplasia represented the largest subgroup, followed by moderate and severe dysplasia. Similar distributions have been reported in studies evaluating OED among high-risk individuals, emphasizing the importance of early screening and timely diagnosis before progression to invasive carcinoma [3,18].

The present study demonstrated a sensitivity of 87.3% and specificity of 81.3% for oral brush cytology. These findings are comparable to those reported by Mehrotra and Gupta [14], who observed sensitivity values ranging from 85% to 92% and specificity values above 80% for oral brush cytology in detecting OED and malignancy. Sciubba [19] also reported high diagnostic reliability of oral brush cytology in identifying premalignant and malignant epithelial alterations. The relatively high sensitivity observed in the current study may be attributed to transepithelial cellular sampling achieved through firm rotational brushing until pinpoint bleeding was observed, thereby ensuring adequate basal and parabasal cell collection.

Toluidine blue staining demonstrated slightly lower sensitivity (82.4%) and specificity (70.8%) compared with oral brush cytology. Similar findings have been reported in studies by Epstein and Guneri [20] and Parakh et al. [12], where toluidine blue staining showed good sensitivity but relatively lower specificity because inflammatory lesions and areas of increased cellular activity may also retain the dye, leading to false-positive results. In the present study, false-positive staining was observed in certain hyperkeratotic and inflammatory lesions, which may explain the reduced specificity of toluidine blue staining compared with oral brush cytology.

Although differences in sensitivity and specificity between the two methods did not achieve statistical significance, oral brush cytology demonstrated significantly better overall diagnostic performance based on AUC and Cohen’s kappa coefficient. The AUC value of 0.843 for oral brush cytology indicated excellent discriminative ability, whereas toluidine blue staining showed comparatively lower diagnostic performance (AUC = 0.766). Furthermore, substantial agreement between oral brush cytology and histopathology (κ = 0.672) was observed, while toluidine blue staining demonstrated only moderate agreement (κ = 0.527). These findings support previous literature, suggesting that brush cytology provides more objective cellular assessment and greater diagnostic reliability than dye-based visualization techniques alone.

Subgroup analysis according to lesion type revealed that both non-invasive methods performed best in erythroplakia lesions, with oral brush cytology demonstrating sensitivity of 95.5%. This finding is clinically relevant because erythroplakia is strongly associated with severe dysplasia and malignant transformation [21]. Lower sensitivity values observed in verrucous lesions may be attributed to excessive keratinization and surface irregularity, which can limit adequate cellular sampling and dye penetration [22]. Similar challenges have been documented in previous studies evaluating adjunctive diagnostic techniques in hyperkeratotic lesions [23].

An important finding of the present study was the improved agreement observed when both oral brush cytology and toluidine blue staining were considered together. Combined positivity yielded higher kappa values, suggesting a potential complementary role for the two techniques in clinical screening protocols. This observation is supported by previous authors who recommended combining adjunctive methods to improve lesion selection and reduce sampling errors during biopsy procedures [5].

The clinical implications of the present study are significant, particularly in resource-limited settings and community-based oral cancer screening programs. Oral brush cytology and toluidine blue staining are simple, minimally invasive, chairside procedures that may aid clinicians in identifying high-risk lesions requiring biopsy and close surveillance. Oral brush cytology, in particular, demonstrated superior overall diagnostic efficacy and may serve as a valuable adjunctive screening tool for early detection of OED and malignancy. Nevertheless, these techniques should not replace histopathological examination, especially in clinically suspicious lesions with persistent symptoms or high-risk features.

Certain limitations of the present study should be acknowledged. The study was conducted at a single center with a relatively limited sample size, which may affect the generalizability of the findings. Interobserver variability in cytological interpretation and dye uptake assessment was not formally evaluated and could have influenced the diagnostic outcomes. In addition, external validation of the findings was not performed, and follow-up evaluation of lesions and long-term assessment of malignant transformation were beyond the scope of the present investigation. Future multicentric longitudinal studies with larger populations, formal assessment of observer agreement, external validation, and incorporation of advanced adjunctive diagnostic technologies may further validate the clinical utility of these non-invasive biopsy procedures.

## Conclusions

Within the limitations of the present study, both oral brush cytology and toluidine blue staining demonstrated satisfactory diagnostic utility for the detection of dysplastic and malignant oral lesions. Oral brush cytology exhibited higher sensitivity, specificity, diagnostic accuracy, and agreement with histopathological findings than toluidine blue staining, suggesting superior diagnostic performance within the study population. The combined use of both techniques may further enhance screening efficiency and lesion assessment. These non-invasive adjunctive methods may serve as valuable chairside aids for the early identification of high-risk oral lesions; however, histopathological examination remains the definitive diagnostic gold standard. Given the single-center nature of the study and the absence of long-term follow-up, further multicenter studies with larger populations are warranted to validate these findings and determine their broader clinical applicability.

## References

[1] Sun R, Dou W, Liu W (2023). Global, regional, and national burden of oral cancer and its attributable risk factors from 1990 to 2019. Cancer Med.

[2] Kumari P, Debta P, Dixit A (2022). Oral potentially malignant disorders: etiology, pathogenesis, and transformation into oral cancer. Front Pharmacol.

[3] Shete M, Kamble PP, Patil K, Bhagawati BT, Brahmankar U, Sharma M (2025). Predictors of dysplasia in oral submucous fibrosis: a retrospective observational study. Cureus.

[4] Ranganathan K, Kavitha L (2019). Oral epithelial dysplasia: classifications and clinical relevance in risk assessment of oral potentially malignant disorders. J Oral Maxillofac Pathol.

[5] Gillenwater A, Papadimitrakopoulou V, Richards-Kortum R (2006). Oral premalignancy: new methods of detection and treatment. Curr Oncol Rep.

[6] Gade LP, Tariq S, Bhukal S (2025). Optimizing oral cancer screening: latent class analysis of chairside adjuncts in a high-risk dental cohort. Cureus.

[7] Idrees M, Farah CS, Sloan P, Kujan O (2022). Oral brush biopsy using liquid-based cytology is a reliable tool for oral cancer screening: a cost-utility analysis: Oral brush biopsy for oral cancer screening. Cancer Cytopathol.

[8] Acha A, Ruesga MT, Rodríguez MJ, Martínez de Pancorbo MA, Aguirre JM (2005). Applications of the oral scraped (exfoliative) cytology in oral cancer and precancer. Med Oral Patol Oral Cir Bucal.

[9] Pallagatti S, Sheikh S, Aggarwal A (2013). Toluidine blue staining as an adjunctive tool for early diagnosis of dysplastic changes in the oral mucosa. J Clin Exp Dent.

[10] Nagaraju K, Prasad S, Ashok L (2010). Diagnostic efficiency of toluidine blue with Lugol's iodine in oral premalignant and malignant lesions. Indian J Dent Res.

[11] Onofre MA, Sposto MR, Navarro CM (2001). Reliability of toluidine blue application in the detection of oral epithelial dysplasia and in situ and invasive squamous cell carcinomas. Oral Surg Oral Med Oral Pathol Oral Radiol Endod.

[12] Parakh MK, Jagat Reddy RC, Subramani P (2017). Toluidine blue staining in identification of a biopsy site in potentially malignant lesions: a case-control study. Asia Pac J Oncol Nurs.

[13] Çelebi E, Öçbe M, Sinanoğlu EA, Tekkeşin MS (2025). Exploring the potential of interdental brush in oral cytology: a pilot study on sampling efficiency. BMC Oral Health.

[14] Mehrotra R, Gupta DK (2011). Exciting new advances in oral cancer diagnosis: avenues to early detection. Head Neck Oncol.

[15] Ge B, Zhu X, Ma M, Ju Y, Ma Y, Song Y (2025). Clinical practice of early screening and risk-stratified management for oral potentially malignant disorders. Front Oncol.

[16] Masthan KM, Babu NA, Sankari SL, Priyadharsini C (2015). Leukoplakia: a short review on malignant potential. J Pharm Bioallied Sci.

[17] van der Waal I, Schepman KP, van der Meij EH, Smeele LE (1997). Oral leukoplakia: a clinicopathological review. Oral Oncol.

[18] Speight PM (2007). Update on oral epithelial dysplasia and progression to cancer. Head Neck Pathol.

[19] Sciubba JJ (1999). Improving detection of precancerous and cancerous oral lesions. Computer-assisted analysis of the oral brush biopsy. U.S. Collaborative OralCDx Study Group. J Am Dent Assoc.

[20] Epstein JB, Güneri P (2009). The adjunctive role of toluidine blue in detection of oral premalignant and malignant lesions. Curr Opin Otolaryngol Head Neck Surg.

[21] Lorenzo-Pouso AI, Lafuente-Ibáñez de Mendoza I, Pérez-Sayáns M, Pérez-Jardón A, Chamorro-Petronacci CM, Blanco-Carrión A, Aguirre-Urízar JM (2022). Critical update, systematic review, and meta-analysis of oral erythroplakia as an oral potentially malignant disorder. J Oral Pathol Med.

[22] Mehrotra D, Goel M, Kumar S, Pandey R, Ram H (2012). Oral verrucous lesions: controversies in diagnosis and management. J Oral Biol Craniofac Res.

[23] Essat M, Cooper K, Bessey A, Clowes M, Chilcott JB, Hunter KD (2022). Diagnostic accuracy of conventional oral examination for detecting oral cavity cancer and potentially malignant disorders in patients with clinically evident oral lesions: systematic review and meta-analysis. Head Neck.

